# Fatty acid synthesis pathway provides lipid precursors for rhamnolipid biosynthesis in *Burkholderia thailandensis* E264

**DOI:** 10.1007/s00253-018-9059-5

**Published:** 2018-05-12

**Authors:** Victor U. Irorere, Thomas J. Smyth, Diego Cobice, Stephen McClean, Roger Marchant, Ibrahim M. Banat

**Affiliations:** 1 School of Biomedical Sciences, Faculty of Life and Health Sciences, Ulster University, Coleraine, Northern Ireland BT52 1SA UK; 2 Department of Life Sciences, Institute of Technology Sligo, County Sligo, Ireland

**Keywords:** Rhamnolipid, FAS II, β-oxidation, *B. thailandensis*, *P. aeruginosa*, *Deuterium labelling*

## Abstract

Rhamnolipid production was monitored for a period of 216 h using different substrates in *Pseudomonas aeruginosa* PAO1 and *Burkholderia thailandensis* E264 which showed comparable crude yields attained by both after 216 h. The crude yield for *P. aeruginosa*, however, was significantly higher at the early stages of fermentation (72 or 144 h). Additionally, *P. aeruginosa* produced rhamnolipid with odd and even carbon chain lipid moieties using odd carbon chain fatty acid substrates (up to 45.97 and 67.57%, respectively). In contrast, *B. thailandensis* produced rhamnolipid with predominantly even carbon chain lipid moieties (up to 99.26). These results indicate the use of the fatty acid synthesis (FAS II) pathway as the main source of lipid precursors in rhamnolipid biosynthesis by *B. thailandensis*. Isotope tracing using 0.25% stearic acid – *d*_*35*_ + 1% glycerol as carbon substrate showed a single pattern of deuterium incorporation: with predominantly less than 15 deuterium atoms incorporated into a single Di-C_14_-C_14_ rhamnolipid molecule. This further indicates that the FAS II pathway is the main source of the lipid precursor in rhamnolipid biosynthesis by *B. thailandensis*. The pathogenicity of these strains was also assessed, and results showed that *B. thailandensis* is significantly less pathogenic than *P. aeruginosa* with an LC50 at 24 h > 2500, approximately three logs higher than *P. aeruginosa* using the *Galleria mellonella* larva model.

**Electronic supplementary material:**

The online version of this article (10.1007/s00253-018-9059-5) contains supplementary material, which is available to authorized users.

## Introduction

The first report of rhamnolipid production was made in the mid twentieth century, with *Pseudomonas aeruginosa* identified as the producer organism. Since then, production of rhamnolipid from strains of *P. aeruginosa* has been extensively investigated to understand the structure, cellular function and mechanism of rhamnolipid production by this organism (Irorere et al. 2017). However, heightened industrial interest has led to an increased demand for rhamnolipids and concerns about the safety of the product and its industrial production processes, since *P. aeruginosa* is an opportunistic human pathogen. These facts have stimulated the search for alternative, safer rhamnolipid producing microorganisms.

In the last decade, a number of *Burkholderia* species have being shown to be capable of rhamnolipid production, including *Burkholderia thailandensis* E264, *Burkholderia pseudomallei*, *Burkholderia glumae* BGR1, *Burkholderia plantarii* and *Burkholderia kururiensis* (Costa et al. 2011; Dubeau et al. 2009; Tavares et al. 2013). The orthologues of the rhamnolipid genes (*rhlA*, *rhlB* and *rhlC*) have been identified in the first three strains (Irorere et al. 2017). Among these strains, *B. thailandensis* E264 is of particular interest mainly due to its significantly lower levels of pathogenicity (Brett et al. 1998; Haraga et al. 2008; Koh et al. 2012).

The production of rhamnolipid by *B. thailandensis* was first reported less than a decade ago. The di-rhamnolipid C_14_-C_14_ was reported as the most abundant congeners produced by this strain, compared to *P. aeruginosa* strains in which di-rhamnolipid C_10_-C_10_ is the most abundant rhamnolipid congener (Dubeau et al. 2009). Also, a genome wide study of *B. thailandensis* E264 showed that the rhamnolipid genes *rhlA*, *rhlB* and *rhlC* are located in a single gene cluster. This cluster is duplicated in the genome of the organism, with both clusters reported to be functioning in rhamnolipid production (Dubeau et al. 2009). This is also different to *P. aeruginosa* in which the *rhlA* and *rhlB* genes are contained in a single operon, while *rhlC* is located in a remote location. Also, these genes are not duplicated in the genome of *P. aeruginosa* (Irorere et al. 2017).

While the production of rhamnolipid has been established in strains of *Burkholderia*, little is known about the mechanism of rhamnolipid biosynthesis in these species. As earlier stated, the majority of what we know about the function and mechanism of biosynthesis of rhamnolipid are obtained from studies using *P. aeruginosa* strains. However, the rhamnolipid congener distribution and the genomic arrangement of the rhamnolipid genes are different in *Burkholderia* compared to *Pseudomonas*. These differences suggest that the metabolism of substrates for rhamnolipid biosynthesis might follow different patterns. To fully exploit these strains as alternatives for industrial rhamnolipid production, it is important to understand their mechanism of rhamnolipid biosynthesis and factors affecting production and composition of the rhamnolipid congeners.

This study was therefore designed to understand the differences in growth and rhamnolipid biosynthesis by *B. thailandensis* in different nutrient limiting media and carbon substrates. Results indicate that the fatty acid synthesis pathway is the main source of the lipid precursor in rhamnolipid biosynthesis in *B. thailandensis*. This differs from *P. aeruginosa* which uses β-oxidation intermediates as the main suppliers of lipid precursors in rhamnolipid biosynthesis (Abdel-Mawgoud et al. 2014).

## Materials and methods

### Microbial strains, media and shake flask fermentation

*Burkholderia thailandensis* E264 (ATCC 700388) and *P. aeruginosa* PAO1 were the strains used in this study (Brett et al. 1998; Stover et al. 2000). Cells were maintained in 50% glycerol at − 80 °C prior to use. Cultures were prepared from freezer stocks in nutrient agar (Sigma Aldrich) plates grown for 24 h at 30 °C. Single colonies were then used to make starter cultures in nutrient broth (Sigma Aldrich) grown overnight at 30 °C with shaking at 200 rpm.

The phosphate-limited media, the proteose-peptone/glucose acid salt media (PPGAS) (Caiazza et al. 2005; Mulligan et al. 1989) and a mineral salt media (MSM) (Moya Ramírez et al. 2015) were used as fermentation media for the production of rhamnolipid. PPGAS comprises Tris-HCl 15 g/l, MgSO_4_·7H_2_O 0.4 g/l, NaCl 1.4 g/l, NH_4_Cl 1 g/l, proteose peptone 10 g/l and carbon source. While MSM comprises NaNO_3_ 2 g/l, Na_2_HPO_4_ 0.9 g/l, KH_2_PO_4_ 0.7 g/l, MgSO_4_·7H_2_O 0.4 g/l, CaCl_2_·2H_2_O 0.1 g/l, trace element solution 0.1% (*v*/*v*) and carbon source. Trace element solution comprises ZnSO_4_·7H_2_O 0.7 g/l, CuSO_4_·5H_2_O 0.5 g/l, MnSO_4_·H_2_O 0.5 g/l, H_3_BO_3_ 0.26 g/l, MoNa_2_O_4_·2H_2_O 0.06 g/l and FeSO_4_·7H_2_O 0.001 g/l. To prevent precipitation during autoclaving, initial preparation of MSM included the carbon source, NaNO_3_, Na_2_HPO_4_ and KH_2_PO_4_. Stock solutions of the other components including the trace elements were prepared, filter sterilised and calculated volumes were added to the autoclaved media. The pH of PPGAS and MSM were adjusted to seven before autoclaving. The addition of the other media components to MSM after autoclaving did not have any significant effect on the final pH of the medium.

Initial shake flask experiments were set up using both PPGAS and MSM prepared as described above with 2% of either oleic acid—C_18_ (BDH chemicals), heptadecanoic acid—C_17_ (Sigma Aldrich, UK) or glycerol (BDH chemical) as carbon source. As heptadecanoic acid and stearic acid are solid powders, after autoclaving but before inoculation, the media were kept in a water bath (Davidson and Harvey Ltd., UK) at 60 °C with shaking at 50 rpm until the white lump formed following autoclaving was dispersed within the medium. The media were then allowed to cool to room temperature before inoculation.

Ten millilitres of overnight starter culture were used to inoculate flasks containing 90 ml fermentation broth to give a 10% inoculum concentration. A sample was taken immediately to determine the initial microbial concentration. The flasks were then incubated at 30 °C for 216 h and samples taken regularly to determine cell growth. All shake flask experiments were performed in triplicate.

Shake flasks experiments were also set up for deuterium tracing studies using MSM as the rhamnolipid production medium supplemented with either 1% glycerol (BDH chemicals) and 0.25% stearic acid (Sigma Aldrich) or 1% glycerol and 0.25% stearic acid – *d*_*35*_ (Sigma Aldrich). Stearic acid – *d*_*35*_ was used in the deuterium tracing studies, as it is readily available compared to oleic acid – *d*_*34*_, while MSM was used due to higher crude rhamnolipid yield compared to PPGAS.

### Determination of microbial growth and biomass concentration

One millilitre of fermentation broth was taken at intervals for determination of viable cell count as described by Miles et al. (1938). Samples were serially diluted, and 20 μl of the diluted samples were inoculated into nutrient agar and incubated for 24 h at 30 °C. Viable cell count was calculated in log_10_ cfu/ml. Analyses were carried out in triplicate for each flask.

To determine biomass concentration, 1 ml of broth was transferred to a preweighed Eppendorf tube and centrifuged for 15 min at 13,000×*g*. The supernatant was discarded, and the pellet washed with PBS, re-centrifuged, and the PBS discarded. Pellets were dried at 80 °C.

### Rhamnolipid extraction and purification

Rhamnolipid extraction was carried out using an acid precipitation and solvent extraction method (Smyth et al. 2014). First, cultures were centrifuged at 13,000×*g* for 15 min. Supernatants were collected and the pH adjusted to 2.0 using 1 M HCl. An equal volume of ethyl acetate (Sigma Aldrich) was then used to extract rhamnolipid from the supernatant three times with the aqueous phase discarded after each extraction. Anhydrous MgSO_4_ (Sigma Aldrich) was added to the organic phase containing the rhamnolipid at a concentration of 0.01 g/ml to remove any residual aqueous phase left after extraction. The ethyl acetate (organic phase) containing the extracted rhamnolipid was filtered using a grade 1 filter paper (Whatman® qualitative filter paper, grade 1) into a round bottom flask and dried under vacuum in a rotary evaporator (Buchi, Flawil, Switzerland) leaving the thick rhamnolipid in the flask. This was then re-dissolved in a small volume of chloroform/methanol (1:1) and transferred into preweighed glass scintillation vials. The samples within the vials were dried using nitrogen gas and the weight of the vials measured until a constant weight was reached.

To remove unwanted impurities and excess carbon source, solid-phase extraction (SPE) of the crude extracts was carried out. This was done using Strata SI-1 Silica (55 μm, 70 A) Giga tubes (Phenomenex). The tubes were conditioned using HPLC-grade chloroform, and the rhamnolipid samples were dissolved in a small amount of chloroform and introduced into the conditioned column. The column was eluted with chloroform until the eluent from the column became clear, indicative of the removal of residual fatty acids and other contaminants. A chloroform/methanol solution in the ratio of 5:0.3 and 5:0.5 was then used to elute the mono-rhamnolipid followed by a 1:1 solution of chloroform/methanol to elute the di-rhamnolipids. Both mono- and di-rhamnolipid were collected together in a preweighed scintillation vial and dried under nitrogen gas.

### Surface tension measurements and determination of the critical micelle concentration

Surface tension was measured using a KRUSS KI0ST Tensiometer with a platinum ring. The surface tension values of a series of rhamnolipid concentrations in water were measured to estimate the critical micelle concentration (CMC). The pH of rhamnolipid samples from *B. thailandensis* E264 was adjusted to seven, to allow the rhamnolipid to completely dissolve in water. Surface tension of each concentration was taken in triplicate and a graph of surface tension against the corresponding concentration was plotted. CMC was determined by extrapolating the intercept of the two sections of the graph.

### Rhamnolipid analysis by LC-QToF-MS

SPE-purified rhamnolipid extracts were characterised by liquid chromatography-hybrid quadrupole time-of-flight mass spectrometry (LC-QToF-MS) using methods described previously (Funston et al. 2016; Smyth et al. 2010b). The Agilent Poroshell SB-C3, 2.1 × 100 mm, particle size 2.7 μm column was used as the static phase to analyse rhamnolipid from *B. thailandensis* E264 while the Poroshell 120, EC-C18, 2.1 × 100 mm, 2.7 μm was used as static phase to analyse rhamnolipids from *P. aeruginosa*. Mobile phase 1 consisted of HPLC grade water (4 mM ammonium acetate) while mobile phase 2 was acetonitrile.

### Pathogenicity assay using *Galleria mellonella*

For *Galleria mellonella* analysis, single colonies of respective organisms were incubated overnight at 30 °C and 200 rpm in nutrient broth (NB). Fifty millilitres of the overnight cultures were centrifuged, and cell pellets were washed in PBS, centrifuged and re-suspended in PBS. Cells were diluted to OD_600_ of 0.46 (7.5 × 10^8^ cfu/ml) and 0.28 (1.35 × 10^9^ cfu/ml) for *B. thailandensis* E264 and *P. aeruginosa* PAO1, respectively. These were further diluted by serial dilution to make working stocks for each individual organism. The cell concentrations of the working stocks were confirmed by plate count. Both the initial stocks and the working stocks were prepared daily and used immediately after preparation.

### Rearing and infection of *G*. *mellonella*

*Galleria mellonella* were purchased from Pets at Home (Coleraine, Northern Ireland, UK) and maintained in the dark on woodchips at 15 °C prior to use. Bacteria suspensions were diluted to known concentration in PBS, and 20-μl samples (100 cfu) were injected into *G. mellonella* via the foremost proleg. Ten larvae were injected for each strain of bacterium, and larvae were incubated for 48 h at 37 °C in perforated Petri dishes to provide adequate ventilation. Larvae injected with 20 μl of sterile PBS were used as a control, and the numbers of dead larvae were scored periodically. Larvae were considered dead when they turn grey/black and did not move in response to a gentle physical stimulation.

To determine if the death of *G. mellonella* is due to the pathogenic effect of the infecting cells or toxicity of the cell components, larvae were also injected with heat-killed cells at similar concentrations (i.e. 100 cfu in 20-μl samples). Cells were heat killed at 80 °C for 2 h in a block heater (Stuart SBH200D, Staffordshire, UK). Cells were confirmed as dead if no growth was observed in an overnight culture of 100-μl sample spread plated on nutrient agar.

To determine the lethal cell number (LC50) of each strain at 24-h post infection, different cell numbers of respective strains were used to inject *G. mellonella* larvae and the number of dead cells was scored at 24 h post infection. The LC50 is the concentration that corresponded to 50% of the larvae death after 24 h, and this was determined by plotting a graph of cell concentration vs percentage death.

### Statistical analysis

All statistical analyses were carried out using GraphPad Prism version 6. Difference in rhamnolipid crude yield from various carbon sources was analysed using two-way ANOVA with Tukey’s multiple comparison (*P* ≥ 0.05). While differences in biomass and rhamnolipid concentration in the deuterium studies were analysed by Mann-Whitney unpaired *t* test.

## Results

### Microbial growth and rhamnolipid production by *B. thailandensis* and *P. aeruginosa*

**Fig. 1:**
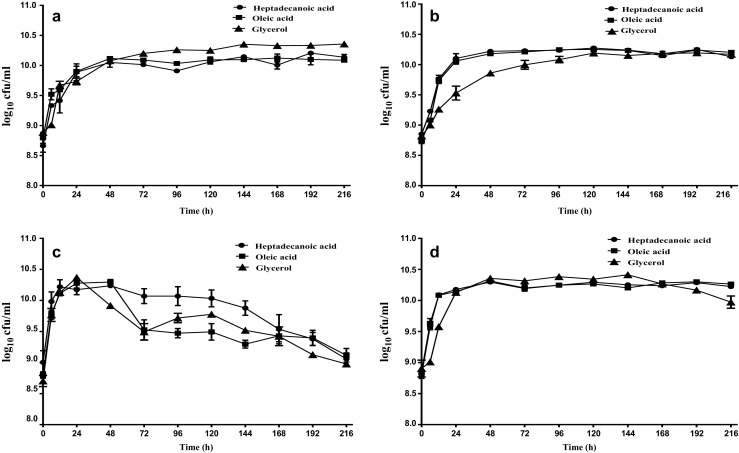
Growth curve of *B. thailandensis* E264 in PPGAS (**a**) or MSM (**b**) and *P. aeruginosa* PAO1 in PPGAS (**c**) or MSM (**d**) under different substrate conditions. Viable counts were carried out following serial dilutions of samples collected at specific time intervals and results obtained were used to construct the growth curve

For rhamnolipid production, both *B. thailandensis* and *P. aeruginosa* were grown for a period of 216 h at 30 °C in MSM or PPGAS media supplemented with different carbon sources. Figure 1 shows the growth curve of both organisms in media supplemented with glycerol, oleic acid or heptadecanoic acid. No significant difference was observed in the initial average cell concentration across and within bacteria strains used in this experiment, with an average initial cell concentration of approximately log_10_ 8.8 cfu/ml. In both media, *B. thailandensis* reached an early stationary phase at 24 h and a late stationary phase at 72 h in media supplemented with fatty acids, or 120 h in media supplemented with glycerol. It maintained this phase until 216 h when fermentation was stopped (Fig. 1a, b). These results correlated with the biomass concentrations which either showed no difference between 72 and 216 h or increased until 216 h, depending on the carbon substrate (Figure S1). On the other hand, *P. aeruginosa* reached early stationary phase around 12 h and went into the late stationary phase between 24 and 48 h. In PPGAS, this phase lasted for a maximum of 72 h with a decline phase observed at 120 h in all carbon substrates (Fig. 1c). In MSM, cells supplemented with fatty acids maintained the late stationary phase until the end of fermentation while cells supplemented with glycerol went into lag phase at 168 h (Fig. 1d).

**Fig. 2:**
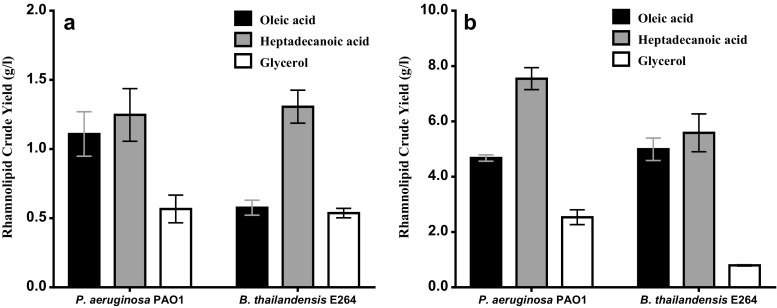
Rhamnolipid crude yield for *P. aeruginosa* and *B. thailandensis* in PPGAS (**a**) and MSM (**b**) using different carbon substrates. Rhamnolipids were extracted from cell-free supernatants by acid precipitation at pH 2, followed by 3× solvent extraction with ethyl acetate

Triplicate extractions were carried out for each fermentation medium and carbon source after 216 h of fermentation to assess the crude yield of rhamnolipid. Results showed a rhamnolipid crude yield of 1.306 and 0.537 g/l by *B. thailandensis* in PPGAS using either heptadecanoic acid or glycerol as carbon source respectively (Fig. 2a). These were comparable to results obtained with *P. aeruginosa* under similar conditions, although the crude yield by *P. aeruginosa* was significantly higher (*P* ≥ 0.05) than *B. thailandensis* using oleic acid as a carbon source in PPGAS (Fig. 2a). Rhamnolipid crude yield in MSM was observed to be considerably higher (*P* ≥ 0.05) compared to PPGAS from all three carbon sources used in the study. The highest yields by *P. aeruginosa* and *B. thailandensis* in MSM were obtained using heptadecanoic acid as a source of carbon (7.543 and 5.588 g/l, respectively). With oleic acid as carbon substrate, the rhamnolipid yield by *B. thailandensis* was 4.99 g/l comparable to a yield of 4.67 g/l in *P. aeruginosa* at the end of the fermentation (Fig. 2b).

**Fig. 3:**
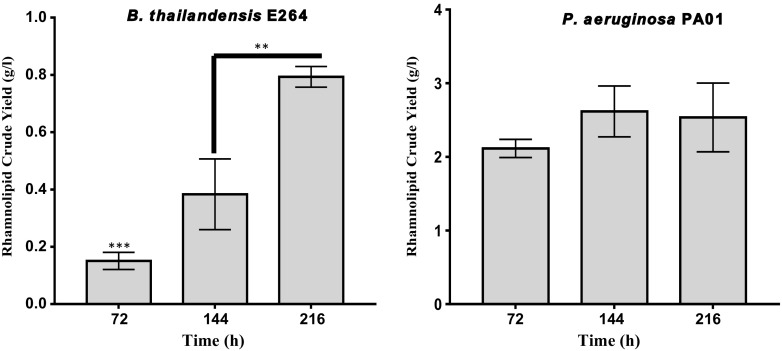
Rhamnolipid crude yield from MSM at different time intervals using glycerol as carbon substrate. Rhamnolipids were extracted from cell-free supernatants by acid precipitation at pH 2, followed by 3× solvent extraction with ethyl acetate; **P < 0.005; ***P < 0.0005

From the growth analysis, it was observed that the growth pattern of *B. thailandensis* is different from *P. aeruginosa*. It had a steady stationary phase until the end of fermentation in all media and carbon sources compared to *P. aeruginosa* which went into decline prior to the end of fermentation. To see if rhamnolipid yield followed a similar pattern to microbial growth, RL extraction was carried out at 72, 144 and 216 h from MSM supplemented with glycerol. Results showed significant increases in rhamnolipid production by *B. thailandensis* with crude yields of 0.15, 0.38 and 0.79 g/l at 72, 144 and 216 h (Fig. 3). In contrast, no significant increase in rhamnolipid production by *P. aeruginosa* was observed at all three extraction times (Fig. 3).

The rhamnolipid obtained from *P. aeruginosa* using either heptadecanoic acid or oleic acid as substrate both had CMCs of 60 mg/l with surface tensions of 31.63 ± 0.06mN/m and 30.30 ± 0.17mN/m respectively at the CMC concentrations. While rhamnolipids from *B. thailandensis* E264 using either heptadecanoic acid or oleic acid as substrate had CMCs of 250 mg/l with surface tensions of 39.16 ± 0.47and 41.90 ± 0.40, respectively.

### Congener distribution of rhamnolipid from different carbon sources in *B. thailandensis* and *P. aeruginosa*

To compare the differences in rhamnolipid congener distribution from the different carbon sources and media, LC-QToF-MS was carried out. Crude extracts were purified by solid-phase extraction prior to analysis. As expected, the results showed that the di-rhamnolipid C_14_-C_14_ with a pseudomolecular ion of 761 *m*/*z* was the most abundant rhamnolipid congener produced by *B. thailandensis* (Table S1). The pseudomolecular ion of 649 *m*/*z* corresponding to di-rhamnolipid C_10_-C_10_ was observed as the most abundant congener produced by *P. aeruginosa* across all carbon sources (Table S2). As expected, the chromatogram of extracts from *P. aeruginosa* cultures supplemented with heptadecanoic acid, showed additional peaks at pseudomolecular ion values of 461, 489, 517, 607, 635 and 663 *m*/*z*. These are indicative of the following odd carbon chain rhamnolipid congeners: mono-C_8_-C_9_/C_9_-C_8_, C_9_-C_10_/C_10_-C_9_, C_10_-C_11_/C_11_-C_10_ and their corresponding di-rhamnolipid congeners, respectively (Table S2). Interestingly, corresponding odd carbon chain rhamnolipid congeners were less obvious in *B. thailandensis* cultures supplemented with heptadecanoic acid (Table S1).

Fragmentation of the parent ion obtained from LC-QToF-MS was carried out for each sample using tandem mass spectrometry (MS/MS) to differentiate isomeric congeners that cannot be resolved chromatographically such as mono or di-C_10_-C_12_/C_12_-C_10_/C_11_-C_11_. These results were used to compare the relative proportion of odd and even carbon chain length rhamnolipids produced with heptadecanoic acid as carbon substrate summarised in Table 1. With heptadecanoic acid as a carbon source, *P. aeruginosa* produced rhamnolipid with odd carbon chain lipid moieties with a cumulative relative abundance of 32.43 and 45.97% in MSM and PPGAS cultures, respectively. In contrast, *B. thailandensis* cultures supplemented with heptadecanoic acid were observed to produce significantly lower quantities of rhamnolipids with odd carbon chain lipid moieties. The cumulative relative abundance of odd carbon chain length rhamnolipids produced by *B. thailandensis* are 0.76 and 2.95% in MSM and PPGAS cultures, respectively (Table 1).

**Table 1:** Composition of rhamnolipid congeners with odd carbon chain lipid moieties from *P. aeruginosa* PAO1 and *B. thailandensis* E264 using heptadecanoic acid as a carbon substrate

Organism	Media	Percentage rhamnolipid with odd chain lipid moieties	
		Odd chain	Even chain
*P. aeruginosa* PAO1	MSM	32.43	67.57
	PPGAS	45.97	54.16
*B. thailandensis* E264	MSM	0.76	99.26
	PPGAS	2.95	97.05

The most abundant odd carbon chain rhamnolipid congeners produced by *P. aeruginosa* in both MSM and PPGAS were di-C_9_-C_10_/C_10_-C_9_ and di-C_10_-C_11_/C_11_-C_10_. Di-C_14_-C_15_/C_15_-C_14_ was the only odd carbon chain rhamnolipid congener produced by *B. thailandensis* from MSM while di-C_13_-C_14_/C_14_-C_13_ was observed in addition in PPGAS (result not shown).

### Growth of *B. thailandensis* in deuterated stearic acid supplemented media

The results observed from the biosynthesis of rhamnolipid using different fatty acid substrates suggest that the lipid component in rhamnolipid biosynthesis is supplied by the fatty acid synthesis pathway (FAS II) and not β-oxidation in the presence of a fatty acid substrate. To investigate this further, we designed an experiment following the procedure described by Zhang et al. (2012) but replacing glucose with glycerol. We supplemented MSM with 1% glycerol and 0.25% stearic acid – *d*_*35*_. A control experiment supplementing MSM with 1% glycerol and 0.25% undeuterated stearic acid was also set up. The hypothesis was that if β-oxidation supplies the lipid precursor in rhamnolipid biosynthesis, then rhamnolipid with at least seven fully deuterated carbons will be observed in the mass spectrometry. This is as described previously in *Pseudomonas* cultures using stearic acid – *d*_*35*_ as co-substrate (Zhang et al. 2012). However, if FAS II supplies the lipid precursor then there will be random incorporation of deuterium within the lipid moieties of the synthesised rhamnolipid.

**Fig. 4:**
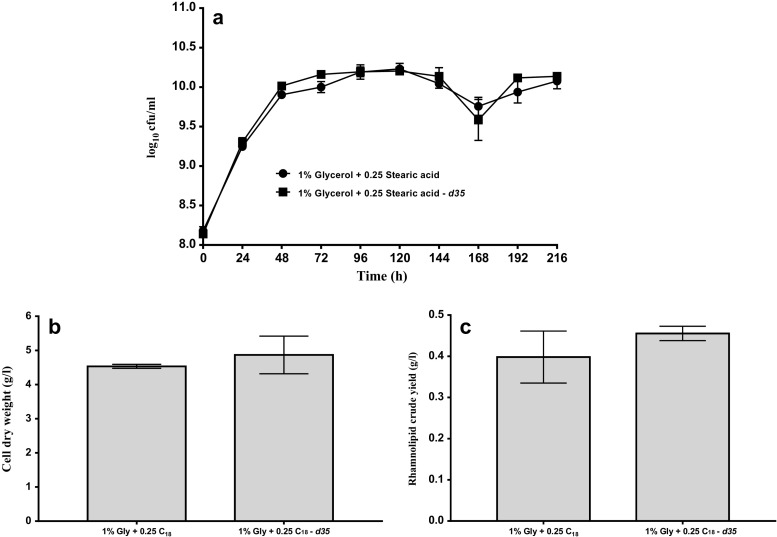
*B. thailandensis* E264 growth curve (**a**), cell dry weight at 216 h (**b**) and rhamnolipid crude yield at 216 h (**c**) in MSM media using either 1% glycerol + 0.25% stearic acid (C_18_) or 1% glycerol + 0.25% stearic acid (C_18_) – *d*_*35*_ as carbon substrate. Viable counts were carried out following serial dilutions of samples collected at specific time intervals, biomass concentration was determined by drying cell pellets at 80 °C to constant weight, rhamnolipid crude extracts were obtained by acid precipitation followed by solvent extraction with ethyl acetate 3×

First, we assessed the impact of using deuterated fatty acid as co-substrate in rhamnolipid biosynthesis on the growth and rhamnolipid production of *B. thailandensis* E264. As seen in Fig. 4a, deuterated and undeuterated stearic acid supplemented media had no significant differences in microbial concentration at each time point during the growth of *B. thailandensis*. This indicates that deuterated stearic acid does not have any effect on the growth of *B. thailandensis*. The biomass concentration was also assessed after 216 h of fermentation. No significant difference in biomass concentration was observed at 216 h of fermentation using either deuterated or undeuterated stearic acid as co-substrate (Fig. 4b). Additionally, no significant difference was observed in the yield of rhamnolipid from deuterated or undeuterated stearic acid as co-substrate (Fig. 4c). Put together, these results suggest that the use of deuterated stearic acid as co-substrate did not have any significant effect on the growth and rhamnolipid biosynthesis of *B. thailandensis*.

### Characterisation of rhamnolipid from deuterated and undeuterated stearic acid

The composition of purified rhamnolipid extracts from deuterated and undeuterated fatty acid is presented in Table 2. The relative abundance is expressed in percentage, and the natural contributions of ^13^C have being corrected in the relative abundance values are presented in Table 2.

**Table 2:** The relative abundance (%) of rhamnolipid congeners with and without deuterium incorporation produced by *B. thailandensis* E264 grown in MSM media supplemented with either 1% glycerol + 0.25% stearic acid or 1% glycerol + 0.25% stearic acid – *d*_*35*_ as carbon substrates

Carbon source	Congener identification	Pseudomolecular ion	Relative abundance (%)
1% glycerol and 0.25% stearic acid	RHA-C_14_-C_14_	615	2.32
	RHA-RHA-C_14_	535	11.04
	RHA-RHA-C_12_-C_12_	705	0.82
	RHA-RHA-C_12_-C_14_/C_14_-C_12_	733	7.38
	RHA-RHA-C_14_-C_14_	761	69.65
	RHA-RHA-C_14_-C_16_/C_16_-C_14_	789	8.77
1% glycerol + 0.25% stearic acid – *d*_*35*_	RHA-C_14_-C_14_	615	1.58
	RHA-C_14_-C_14_ – *d*_*1*_	616	0.67
	RHA-C_14_-C_14_ – *d*_*2*_	617	0.53
	RHA-C_14_-C_14_ – *d*_*3*_	618	0.42
	RHA-C_14_-C_14_ – *d*_*4*_*, d*_*5*_*, d*_*6*_*, d*_*7*_	619, 620, 621, 622	0.17, 0.18, 0.22, 0.11
	RHA-RHA-C_14_	535	3.60
	RHA-RHA-C_14_ – *d*_*1*_	536	1.87
	RHA-RHA-C_14_ – *d*_*2*_	537	1.57
	RHA-RHA-C_14_ – *d*_*3*_	538	0.87
	RHA-RHA-C_14_ – *d*_*4*_	539	0.57
	RHA-RHA-C_14_ – *d*_*5*_*, d*_*6*_	540, 541	0.32, 0.13
	RHA-RHA-C_12_-C_12_	705	0.78
	RHA-RHA-C_12_-C_12_ – *d*_*1*_	706	0.45
	RHA-RHA-C_12_-C_12_ – *d2*	707	0.41
	RHA-RHA-C_12_-C_12_ – *d*_*3*_*, d*_*4*_*, d*_*5*_*, d*_*7*_	708, 709, 710, 712	0.15, 0.13, 0.11, 0.15
	RHA-RHA-C_12_-C_14_/C_14_-C_12_	733	3.95
	RHA-RHA-C_12_-C_14_/C_14_-C_12_ – *d*_*1*_	734	2.25
	RHA-RHA-C_12_-C_14_/C_14_-C_12_ – *d*_*2*_	735	2.16
	RHA-RHA-C_12_-C_14_/C_14_-C_12_ – *d*_*3*_	736	1.17
	RHA-RHA-C_12_-C_14_/C_14_-C_12_ – *d*_*4*_	737	0.77
	RHA-RHA-C_12_-C_14_/C_14_-C_12_ – *d*_*5*_	738	0.89
	RHA-RHA-C_12_-C_14_/C_14_-C_12_ – *d*_*6*_	739	0.76
	RHA-RHA-C_12_-C_14_/C_14_-C_12_ – *d*_*7*_*, d*_*8*_*, d*_*9*_*, d*_*10*_*, d*_*11*_	740, 741, 742, 743, 744	0.64, 0.37, 0.18, 0.24, 0.12
	RHA-RHA-C_14_-C_14_	761	15.28
	RHA-RHA-C_14_-C_14_ – *d*_*1*_	762	9.02
	RHA-RHA-C_14_-C_14_ – *d*_*2*_	763	8.32
	RHA-RHA-C_14_-C_14_ – *d*_*3*_	764	5.43
	RHA-RHA-C_14_-C_14_ – *d*_*4*_	765	4.50
	RHA-RHA-C_14_-C_14_ – *d*_*5*_	766	3.88
	RHA-RHA-C_14_-C_14_ – *d*_*6*_	767	3.77
	RHA-RHA-C_14_-C_14_ – *d*_*7*_*, d*_*8*_*, d*_*9*_*, d*_*10*_*, d*_*11*_*, d*_*12*_*, d*_*13*_*, d*_*14*_*, d*_*15*_	768, 769, 770, 771, 772, 773, 774, 775, 776	2.88, 2.26, 1.91, 1.23, 0.89, 0.46, 0.38, 0.11, 0.06
	RHA-RHA-C_14_-C_16_/C_16_-C_14_	789	2.29
	RHA-RHA-C_14_-C_16_/C_16_-C_14_ – *d*_*1*_	790	1.29
	RHA-RHA-C_14_-C_16_/C_16_-C_14_ – *d*_*2*_	791	1.58
	RHA-RHA-C_14_-C_16_/C_16_-C_14_ – *d*_*3*_	792	0.89
	RHA-RHA-C_14_-C_16_/C_16_-C_14_ – *d*_*4*_	793	0.70
	RHA-RHA-C_14_-C_16_/C_16_-C_14_ – *d*_*5*_*, d*_*6*_*, d*_*7*_*, d*_*8*_*, d*_*9*_*, d*_*10*_*, d*_*11*_*, d*_*12*_*, d*_*13*_	794, 795, 796, 797, 798, 799, 800, 801, 802	0.68, 0.67, 0.53, 0.62, 0.44, 0.23, 0.21, 0.20, 0.09

In cultures treated with undeuterated stearic acid as co-substrate, six major rhamnolipid congeners were identified: mono-C_14_-C_14_, di-C_14_, di-C_12_-C_12_, di-C_12_-C_14_/C_14_-C_12_, di-C_14_-C_14_ and di-C_14_-C_16_/C_16_-C_14_ (Table 2). In media containing deuterated stearic acid, these six congeners were also identified; however, additional peaks corresponding to various rates of deuterium incorporation were also found. In all the six major congeners, there is a progressive decline in the number of deuterium atom incorporation, with single deuterium incorporation having the highest percentage abundance across all congeners. Additionally, the maximum amount of deuterium incorporation observed was 15, seen only in the di-C_14_-C_14_ at a very low abundance of 0.06% (Table 2).

### Pathogenicity of *B. thailandensis* E264 and *P. aeruginosa* PAO1

**Fig. 5:**
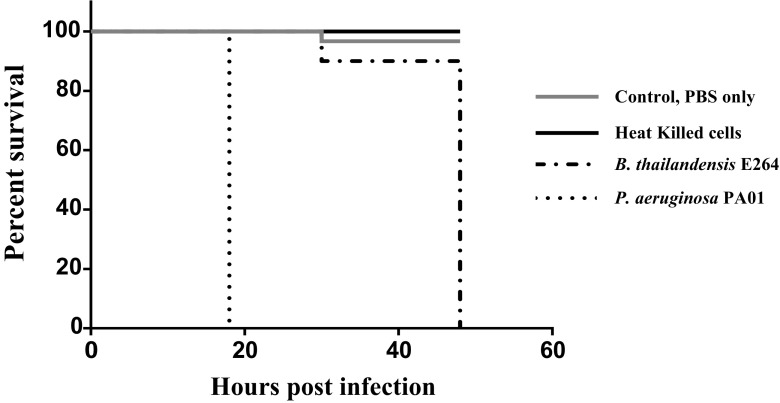
Kaplan-Meier plot of percentage survival of *Galleria mellonella* larvae after infection with 100 cfu of live or heat-killed cells of *P*. *aeruginosa* PAO1 and *B. thailandensis* E264. Heat-killed cells shown are for both strains and did not result in mortality at 48 h post infection while the negative control is sterile PBS in which 1 death of 30 was recorded at 30 h post infection. *n* = 30 (pooled from triplicate experiments each with 10 larvae)

Using the *G. mellonella* model, we compared the pathogenicity of *B. thailandensis* and *P. aeruginosa*. Results showed 100% mortality in larvae infected with 100 cfu of *P. aeruginosa* within 24 h of infection (Fig. 5). However, infecting larvae with 100 cfu of *B. thailandensis* showed 100% survival at 24 h. Although, mortality rate increases after 24 h and a 100% mortality can be observed at 48 h post infection. The LC50 of both organisms at 24 h was determined and results showed that *P. aeruginosa* has LC50 of < 3.5 cfu while *B. thailandensis* has an LC50 of approximately 2500 cfu. This indicates that the LC50 of *B. thailandensis* at 24 h in *G. mellonella* is approximately three logs higher than that of *P. aeruginosa*. No mortality was observed using heat-killed cells of either organism (Fig. 5).

## Discussion

The two major studies on rhamnolipid production by *B. thailandensis* E264 were carried out in nutrient rich broth, supplemented with either glycerol or canola oil as carbon sources (Dubeau et al. 2009; Funston et al. 2016). In the latter study, it was shown that the organism had a sustained stationary phase until the end of fermentation (Funston et al. 2016). Our study confirms that *B. thailandensis* has a sustained stationary phase even when grown in minimal or nutrient limiting conditions, with no decline phase observed in its growth curve during the period of this study. However, *P. aeruginosa* went into decline phase at all carbon substrate conditions in PPGAS and with glycerol as substrate in MSM (Fig. 1). We chose *P. aeruginosa* PAO1 for this comparative study, as it has been shown to be a model for the study of rhamnolipid production (Müller et al. 2010).

We have earlier shown that the rhamnolipid genes (*rhlA*, *rhlB* and *rhlC*) are expressed at elevated levels throughout the stationary phase of growth in *B. thailandensis* (Funston et al. 2016). This is not the case in *P. aeruginosa* where *rhlA* and *rhlB* are downregulated as the cells enter into stationary growth phase with *rhlC* expression elevated at the onset of stationary phase and then declining subsequently (Perfumo et al. 2013). An extended stationary phase coupled with continuous expression of rhamnolipid synthesis genes will potentially result in continuous rhamnolipid production. This is because the rate of rhamnolipid production is known to increase as the cells enter their stationary phase (Benincasa et al. 2002). This may therefore account for the continuous increase in rhamnolipid yield, observed throughout the period of fermentation in *B. thailandensis*, compared to *P. aeruginosa* where the rhamnolipid yield at 72 h was comparable to that at 216 h (Fig. 3).

Additionally, no significant difference in rhamnolipid crude yield from *B. thailandensis* was observed at 216 h compared to *P. aeruginosa* in either PPGAS (with either heptadecanoic acid or glycerol as carbon source) or MSM (with oleic acid as carbon source). Given that these media and growth conditions were designed for rhamnolipid production in *P. aeruginosa*, it is possible that optimising production conditions for *B. thailandensis* can give yields with values higher than those observed in *P. aeruginosa*. However, it is important to note that while rhamnolipid crude yield in *B. thailandensis* can be comparable to that from *P. aeruginosa* at 216 h, crude yield in *P. aeruginosa* peaked at 72 h in this study or at 48 h as reported in previous comparative studies (De-Rienzo et al. 2016). At this time (72 h), the rhamnolipid crude yield from *P. aeruginosa* was significantly higher compared to *B. thailandensis* under all conditions studied. As the industrial cost of running a fermenter for 216 h is substantially higher than that needed to run the same fermentation for 72 h, industries would prefer to use *P. aeruginosa* in their rhamnolipid production; considering cost savings only. However, the development of methods to enhance the early initiation of rhamnolipid production in *B. thailandensis* will further boost the industrial appeal of this organism as an alternative rhamnolipid producer. For example, a study in 2010 with *P. aeruginosa* showed that iron limitation resulted in the expression of *rhlA*, seven- to tenfold earlier compared to iron rich media and this simultaneously led to early initiation of rhamnolipid production (Glick et al. 2010). Also, the introduction of exogenous quorum sensing molecules is known to induce and increase the production of rhamnolipid in *P. aeruginosa* (Dusane et al. 2010). Similar techniques as those previously can be applied to induce the synthesis of rhamnolipid early in the growth of *B. thailandensis*. Thus, improving the overall yield of rhamnolipid from these strains and subsequently make them more industrially relevant.

The industrial appeal of *B. thailandensis* is further boosted by its significantly lower pathogenicity confirmed in this study. *B. thailandensis* E264 is a biosafety level 1 organism and is generally regarded to be non-pathogenic. However, its physiological similarity with the pathogenic *B. pseudomallei*, the causative agent of melioidosis, coupled with a recent report of disease caused by infection with strains of *B. thailandensis* (not E264) (Glass et al. 2006) led us to compare the pathogenicity of *P. aeruginosa* PAO1 and *B. thailandensis* E264. As expected, results using the *G. mellonella* virulence model showed that *B. thailandensis* E264 is a less pathogenic alternative for rhamnolipid production compared to *P. aeruginosa* PAO1 (Fig. 5). The LC50 at 24 h of *B. thailandensis* was also shown to be three logs higher than that of *P. aeruginosa*. Furthermore, no lethal effect of heat-killed cells was observed in both strains ruling out the possibility of cell toxicity rather than pathogenicity.

One of the main objective of this study was to analyse the differences is congener distribution produced by *B. thailandensis* and *P. aeruginosa* when grown under different substrate conditions. Results showed that *B. thailandensis* produces rhamnolipid with longer chain length lipid moieties (predominantly C_14_-C_14_) and fewer congeners (< 15 including isomeric congeners). While *P. aeruginosa* produces shorter chain length rhamnolipid lipid moieties (predominantly C_10_-C_10_), with more diverse congener distribution (> 36 different congeners identified in this study). These results corroborate previous studies (De-Rienzo et al. 2016; Dubeau et al. 2009; Elshikh et al. 2017; Funston et al. 2016).

However, no report on the effect of fatty acid substrate chain length on the rhamnolipid lipid moieties of *B. thailandensis* is available in the literature. It has previously been reported that when *P. aeruginosa* is fed with odd carbon chain fatty acid as a carbon substrate, the cells produce rhamnolipids with odd and even carbon chain lipid components. While cells fed with even chain fatty acids produce rhamnolipid with purely even carbon chains (Hori et al. 2011). This led to the assumption that the lipid component of rhamnolipid is either supplied by β-oxidation or that β-oxidation intermediates are diverted into the fatty acid synthesis pathway (FAS II) and subsequently used as lipid precursors for rhamnolipid biosynthesis. The latter assumption was later proved by Zhang et al. using isotope tracing and gene expression studies of both β-oxidation and FAS II synthesis genes (Zhang et al. 2012). This is also backed by previous studies that have shown that *P. aeruginosa* is able to shunt intermediates of β-oxidation into the FAS II pathway (Yuan et al. 2012). However, a more recent report has shown that β-oxidation directly supplies the lipid precursor in rhamnolipid biosynthesis without the need for FAS II elongation (Abdel-Mawgoud et al. 2014).

To see if either of these is true for *B. thailandensis*, this study assessed the rhamnolipid congener distribution of *B. thailandensis*. Odd or even carbon chain length fatty acids or glycerol were used as carbon source; *P. aeruginosa* was used as a control. As expected, it was observed that when grown with either even chain fatty acid or glycerol, *P. aeruginosa* produces rhamnolipid with solely even carbon chain lipid moieties. While when grown in odd carbon chain fatty acid (C_17_), it produced rhamnolipid with both odd and even carbon chain lipid moieties (Table S2). Similarly, when *B. thailandensis* was grown with even carbon chain fatty acid or glycerol as carbon source, only rhamnolipids with even carbon chain lipid moieties were observed. Interestingly, when *B. thailandensis* was grown with odd carbon chain fatty acid as the sole carbon source, it produces rhamnolipid with predominantly even carbon chain lipid moieties (Table S1). This result was unexpected and suggested that the rhamnolipid lipid moieties of *B. thailandensis* are obtained predominantly from the FAS II pathway. Different from *P. aeruginosa*, known to obtain its lipid precursor predominantly from β-oxidation (Abdel-Mawgoud et al. 2014).

To further investigate this, we carried out isotope tracing studies using 1% glycerol + 0.25% stearic acid – *d*_*35*_ as carbon sources, 1% glycerol + 0.25% stearic acid was used as a control. We first analysed the effect of using deuterated co-substrate in bacteria growth and rhamnolipid production. This was done as previous report has shown that *P. aeruginosa* is inhibited by deuterated substrates (Smyth et al. 2010a) and the organism needed to be ‘trained’ to grow on them. Results indicate that the use of deuterated stearic acid as co-substrate in rhamnolipid biosynthesis did not have any effect on either the growth or rhamnolipid production of *B. thailandensis* (Fig. 4a–c). This could be because deuterated stearic acid is only a co-substrate in this study; therefore, cells can easily adapt to it and grow using glycerol supplied as an additional substrate. This is further evidenced in a previous study that used deuterated stearic acid as co-substrate in rhamnolipid production by *P. aeruginosa*. The report showed that deuterated stearic acid did not have any effect on either microbial growth or rhamnolipid yield (Zhang et al. 2012).

The synthesised rhamnolipid were then characterised to see the pattern of deuterium incorporation within the various congeners. Isotopologous rhamnolipid congeners with varied levels of deuterium incorporation in all congeners were observed. Deuterium incorporation from 0 to 15 can be seen in the most abundant Di-C_14_-C_14_ rhamnolipid congener. To see if FAS II synthesis can explain these various levels of deuterium incorporation, we carried out isotope tracing analysis for the synthesis of (R)-β-hydroxytetradecanoyl-ACP, the lipid precursor for the synthesis of Di-C_14_-C_14_. The analysis showed that in a single C_14_, isotopes ranging from C_14_-*d*_*0*_ to C_14_-*d*_*10*_ are possible (Table S3 and Figure S2). This therefore suggests that a maximum of 20 deuterium atoms can be incorporated into the lipid structure of the Di-C_14_-C_14_ using FAS II lipid precursors.

If β-oxidation of stearic acid – *d*_*35*_ directly supplies the lipid precursor to synthesise Di-C_14_-C_14_, as previously suggested for *P. aeruginosa* (Abdel-Mawgoud et al. 2014), then a minimum of 27 deuterium atoms will be present in a single C_14_ chain, as it will be fully deuterated. This will result in 54 deuterium atoms in a Di-C_14_-C_14_ rhamnolipid congener. Also, if β-oxidation intermediates (C_8_-*d*_*15*_) are diverted to FAS II and elongated to give the C_14_ lipid precursor, as was also suggested for *P. aeruginosa* (Zhang et al. 2012), then a minimum of 15 deuterium atoms will be present in a single C_14_ lipid chain. This will give a minimum number of 30 deuterium atoms in a Di-C_14_-C_14_ rhamnolipid congener.

Based on the explanations mentioned and results from isotope tracing, we suggest that the lipid precursor for rhamnolipid biosynthesis in *B. thailandensis* is obtained from FAS II or de novo fatty acid synthesis. Combining results obtained from studies using different fatty acid substrate and that of isotope tracing, we concluded that FAS II is the main supplier of the lipid precursor in *B. thailandensis*. However, the observation of small quantities of single odd chain lipid moiety in the Di-C_14_-C_14_ using odd chain fatty acid as carbon source suggests the use of either direct β-oxidation or elongated β-oxidation products in rhamnolipid biosynthesis. These, however, contribute less than 3% of the total rhamnolipid yield based on our study. These results suggest that the rhlA of *B. thailandensis* has a preference for β-hydroxylacyl-ACP from FAS II compared to those from β-oxidation. The fact that *B. thailandensis* produces rhamnolipid with long chain lipid moieties (majorly C_12_, C_14_ and C_16_) further supports the supply of lipid precursors by the FAS II pathway. This is because fatty acid biosynthesis in most biological systems is known to be terminated with the C_16_ saturated straight chain palmitic acid (Smith 1994).

The use of FAS II as the main supplier of the lipid precursor in rhamnolipid biosynthesis is industrially relevant. It implies that *B. thailandensis* will be able to use a wide range of substrates with yields comparable to those of fatty acid substrates, compared to *P. aeruginosa* in which yield from sugars are significantly lower than those from lipids. Although in this study, fatty acid substrates produced the highest yields compared to glycerol. This might be because hydroxyfatty acids from β-oxidation are recruited for other cell processes, thus more β-hydroxyfatty acids from FAS II are available for rhamnolipid biosynthesis. This creates an opportunity to genetically modify *B. thailandensis* for increased rhamnolipid production, by creating mutants unable to divert FAS II products to other metabolic cell processes. A clear example is the enhanced rhamnolipid production in polyhydroxyalkanoate (PHA)-deficient mutants of *B. thailandensis* (Funston et al. 2017). Contrarily, PHA-deficient mutants of *P. aeruginosa* did not produce significantly higher rhamnolipid yields compared to wild type; however, rhamnolipid-deficient mutants produced significantly higher yields of PHA (Choi et al. 2011).

In conclusion, these results show that *B. thailandensis* is a potentially less pathogenic substitute for industrial production of rhamnolipid compared to *P. aeruginosa*. However, to fully exploit these strains, it is important that more research be carried out to understand factors that can improve rhamnolipid yield, especially in the initial stages of cell growth as explained earlier. Furthermore, studies of rhamnolipid biosynthesis using different fatty acid substrates coupled with isotope tracing suggest that rhamnolipid biosynthesis in *B. thailandensis* differs significantly from *P. aeruginosa*. This further strengthens the need for studies in rhamnolipid biosynthesis in the *Burkholderia* species, to further understand their natural function and mechanism of biosynthesis. These studies will help improve the industrial appeal of these less virulent strains in rhamnolipid biosynthesis compared to *P. aeruginosa*.

## Electronic supplementary material

ESM 1 (PDF 484kb)
