# Supplementary material for: Fatty acid synthesis pathway provides lipid precursors for rhamnolipid biosynthesis in Burkholderia thailandensis E264
Source: Appl Microbiol Biotechnol. 2018 May 12;102(14):6163–74. doi: 10.1007/s00253-018-9059-5 (PMC6013509; doi:10.1007/s00253-018-9059-5)

APPLIED MICROBIOLOGY AND BIOTECHNOLOGY

SUPPLEMENTARY MATERIALS FOR

Fatty Acid Synthesis Pathway provides lipid Precursors for rhamnolipid biosynthesis in *Burkholderia thailandensis* E264

Victor U. Irorere [1], Thomas J. Smyth [2], Diego Cobice [1], Stephen McClean [1], Roger Marchant [1], Ibrahim M. Banat [1*]

Victor U. Irorere [1] (Irorere-V@ulster.ac.uk)

Thomas J. Smyth [2] (smyth.thomas@itsligo.ie)

Diego Cobice [1] (d.cobice@ulster.ac.uk)

Stephen McClean [1] (s.mcclean@ulster.ac.uk)

Roger Marchant [1] (r.marchant@ulster.ac.uk)

Ibrahim M. Banat [1*] (im.banat@ulster.ac.uk)

[1] School of Biomedical Sciences, Faculty of Life and Health Sciences, Ulster University, Coleraine, BT52 1SA, Northern Ireland, UK.

[2] Department of Life Sciences, Institute of Technology Sligo, County Sligo, Ireland.

*Corresponding author: im.banat@ulster.ac.uk

Table S1: Relative abundance of rhamnolipid congeners produced by *B. thailandensis* E264 grown in MSM media using different carbon substrates. Percentage abundance of the individual rhamnolipid congeners were obtained by fragmentation of the rhamnolipids by tandem mass spectrometry. Odd carbon chain lipid components can be observed using heptadecanoic acid (HDA).

**Rhamnolipid congener**	**Pseudomolecular ion (m/z)**	**Retention time (mins)**	**Relative abundance (%)**		
			Glycerol	***Oleic acid***	***HDA***
RHA-C_{12}-C_{12}	558	6.7	0.15	0.19	0.61
RHA-C_{12}-C_{14}/ C_{14}-C_{12}	586	9	0.67	1.05	3.57
RHA-C_{14}-C_{14}	615	11.2	5.32	8.27	18.84
RHA-RHA-C_{10}-C_{12}/ C_{12}-C_{10}	676	3.1	0.71	0.21	0.31
RHA-RHA-C_{12}-C_{12}	705	5.3	1.13	1.73	2.69
RHA-RHA-C_{12}-C_{14}	733	7.5	0.67	0.56	2.42
RHA-RHA-C_{14}-C_{12}	733	7.5	4.13	6.5	12.72
RHA-RHA- C_{14}-C_{14}	761	9.7	81.45	76.15	52.74
RHA-RHA-C_{14}-C_{15}/ C_{15}-C_{14}	775	10.7	ND*	ND	0.76
RHA-RHA-C_{14}-C_{16}	789	11.7	1.92	2.35	1.61
RHA-RHA-C_{16}-C_{14}	789	11.7	3.91	2.98	3.73

*ND = Below limit of detection

Table S2: Relative abundance of rhamnolipid congeners produced by *P. aeruginosa* PAO1 grown in MSM media using different carbon substrates. Percentage abundance of the individual rhamnolipid congeners were obtained by fragmentation of the rhamnolipids by tandem mass spectrometry (LC/MS/MS). Odd chain lipid components can be observed using Heptadecanoic acid (HDA).

**Rhamnolipid congener**	**Pseudomolecular ion (m/z)**	**Retention time (mins)**	**Relative abundance (%)**		
			Glycerol	***Oleic acid***	***HDA***
RHA-C_8-C_9/ C_9-C_8	461	2.9	ND	ND	0.94
RHA-C_{10}-C_8	475	4.7	1.86	2.51	2.31
RHA-C_8-C_{10}	475	4.7	2.46	1.67	3.47
RHA-C_{10}-C_9	489	7	ND	ND	1.65
RHA-C_9-C_{10}	489	7	ND	ND	4.69
RHA-C_{10}-C_{10}	503	9.3	30.02	35.99	12.91
RHA-C_{10}-$_{11}$	517	11.8	ND*	ND	0.66
RHA-C_{11}-$_{10}$	517	11.8	ND	ND	0.16
RHA-C_{10}-$C_{12:1}$	529	12	1.83	1.47	ND
RHA-$C_{12:1}$-C_{10}	529	12	0.23	0.42	ND
RHA-C_{10}-C_{12}	531	14.2	0.77	0.86	ND
RHA-C_{12}-C_{10}	531	14.2	0.36	0.29	ND
RHA-RHA-C_9-C_8	607	2.3	ND	ND	0.71
RHA-RHA-C_8-C_9	607	2.3	ND	ND	0.56
RHA-RHA-C_{10}-C_8	621	3.3	2.57	1.29	2.9
RHA-RHA-C_8-C_{10}	621	3.3	6.29	2.4	7.35
RHA-RHA-C_9-C_9	621	3.3	ND	ND	1.34
RHA-RHA-C_{10}-C_9	635	5.3	ND	ND	5.44
RHA-RHA-C_9-C_{10}	635	5.3	ND	ND	10.81
RHA-RHA-C_{10}-C_{10}	649	7.5	47.35	48.28	36.6
RHA-RHA-C_{11}-C_{10}	663	9.5	ND	ND	1.49
RHA-RHA-C_{10}-C_{11}	663	9.5	ND	ND	3.64
RHA-RHA-C_{10}-$C_{12:1}$	675	9.8	2.4	1.67	0.89
RHA-RHA-C_{10}-C_{12}	677	11.5	3.1	2.46	0.9
RHA-RHA-C_{12}-C_{10}	677	11.5	0.77	0.7	0.24
RHA-RHA-C_{11}-C_{11}	677	11.5	ND	ND	0.34

*ND = Below limit of detection

Table S3: Isotope tracing analysis for the synthesis of isotopologous (R)-β-hydroxytetradecanoyl-ACP through the FAS II pathway. '×' signifies that there is a chance of the synthesis of the specific isotope. Isotopologous (R)-β-hydroxytetradecanoyl-ACP - d_n can be synthesized with n ranging from 0 to 10. AcetylCoA d_2 can be obtained from the initial stages of beta oxidation while AcetylCoA-d_0 is from glycerol metabolism. We have not shown all possible combinations here but we have shown all combinations that can result in both the minimum and maximum deuterium incorporation and those in between.

**FAS II initiation step (Cycle 1)**		**FAS II elongation steps**					**(R)-β-hydroxytetradecanoyl-ACP (d_n)**										
		**Cycle 2**	**Cycle 3**	**Cycle 4**	**Cycle 5**	**Cycle 6**											
**Acetyl CoA (d_n)**	**Malonyl ACP (d_n)**	**Malonyl ACP (d_n)**	**Malonyl ACP (d_n)**	**Malonyl ACP (d_n)**	**Malonyl ACP (d_n)**	**Malonyl ACP (d_n)**	**d_0**	**d_1**	**d_2**	**d_3**	**d_4**	**d_5**	**d_6**	**d_7**	**d_8**	**d_9**	**d_{10}**
**d_0**	**d_0**	**d_0**	**d_0**	**d_0**	**d_0**	**d_0**	**x**										
						**d_1**		**x**									
						**d_2**			**x**								
**d_1**	**d_1**	**d_0**	**d_0**	**d_0**	**d_0**	**d_2**				**x**	**x**						
		**d_1**	**d_0**	**d_0**	**d_0**	**d_2**				**x**	**x**	**x**					
			**d_1**	**d_0**	**d_0**	**d_2**				**x**	**x**	**x**	**x**				
				**d_1**	**d_0**	**d_2**				**x**	**x**	**x**	**x**	**x**			
					**d_1**	**d_2**				**x**	**x**	**x**	**x**	**x**	**x**		
**d_2**	**d_2**	**d_2**	**d_2**	**d_2**	**d_0**	**d_2**									**x**		
					**d_1**	**d_2**									**x**	**x**	
					**d_2**	**d_2**										**x**	
**d_3**	**d_2**	**d_2**	**d_2**	**d_2**	**d_0**	**d_2**										**x**	
					**d_1**	**d_2**										**x**	**x**
					**d_2**	**d_2**											**x**

Figure S1: Biomass concentration of *B. thailandensis* in PPGAS (a) or MSM (b) and *P. aeruginosa* in PPGAS (c) or MSM (d) at 72, 144, and 216 h of fermentation during shake flask studies in different carbon sources. Each time point is the mean of three replicate experiments with the error bars representative of the standard deviation of the mean.

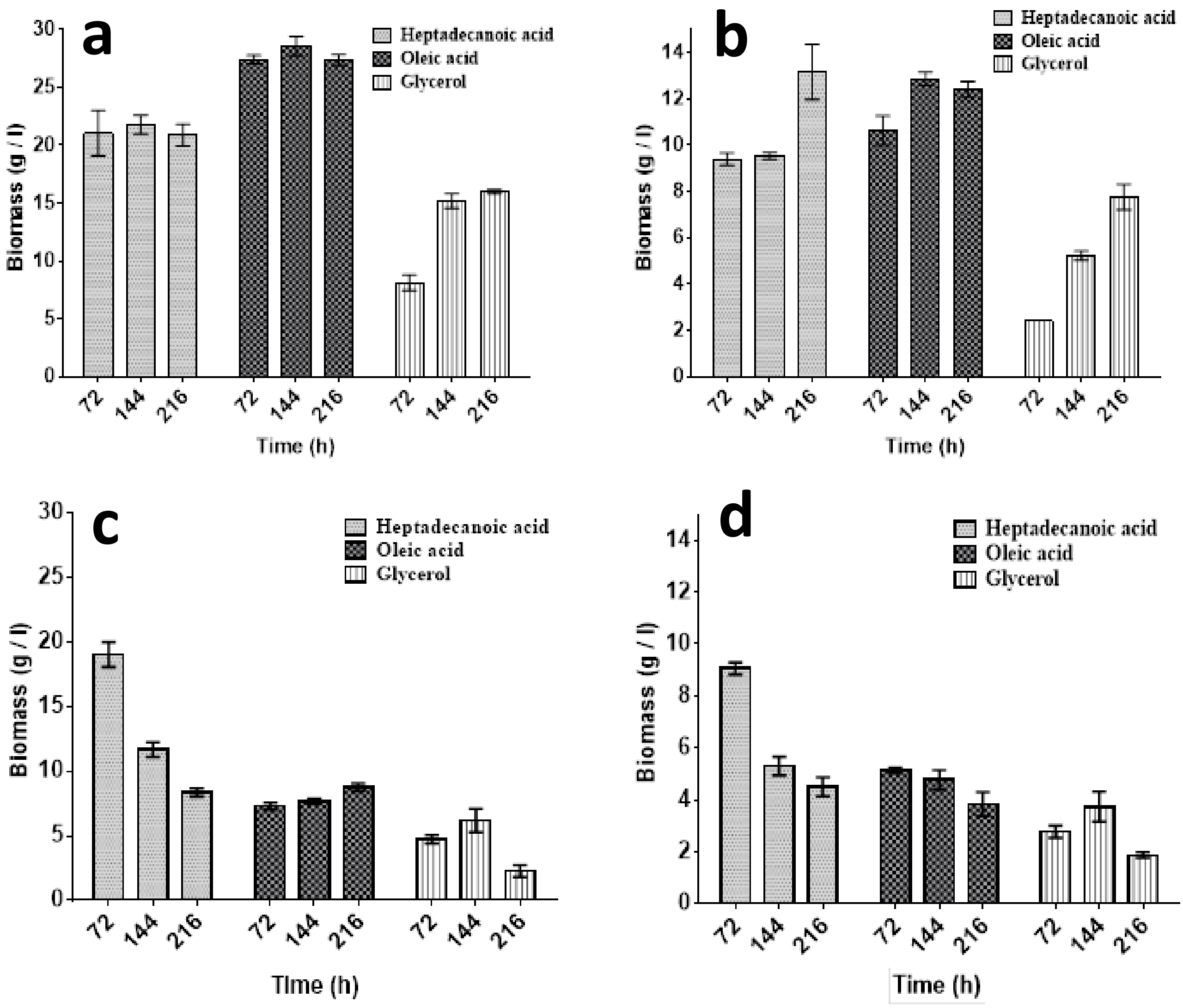

Figure S2: Possible pathway for the synthesis of both di-deuterated **(a)** mono-deuterated **(b)** undeuterated (**c**) malonylACP and undeuterated acetyl CoA (**d**) from either deuterated acetylCoA obtained from β-oxidation of deuterated fatty acid or undeuterated acetylCoA obtained from glycerol metabolism. Deuterated acetylCoA can also form deuterated acetylACP (process not shown).

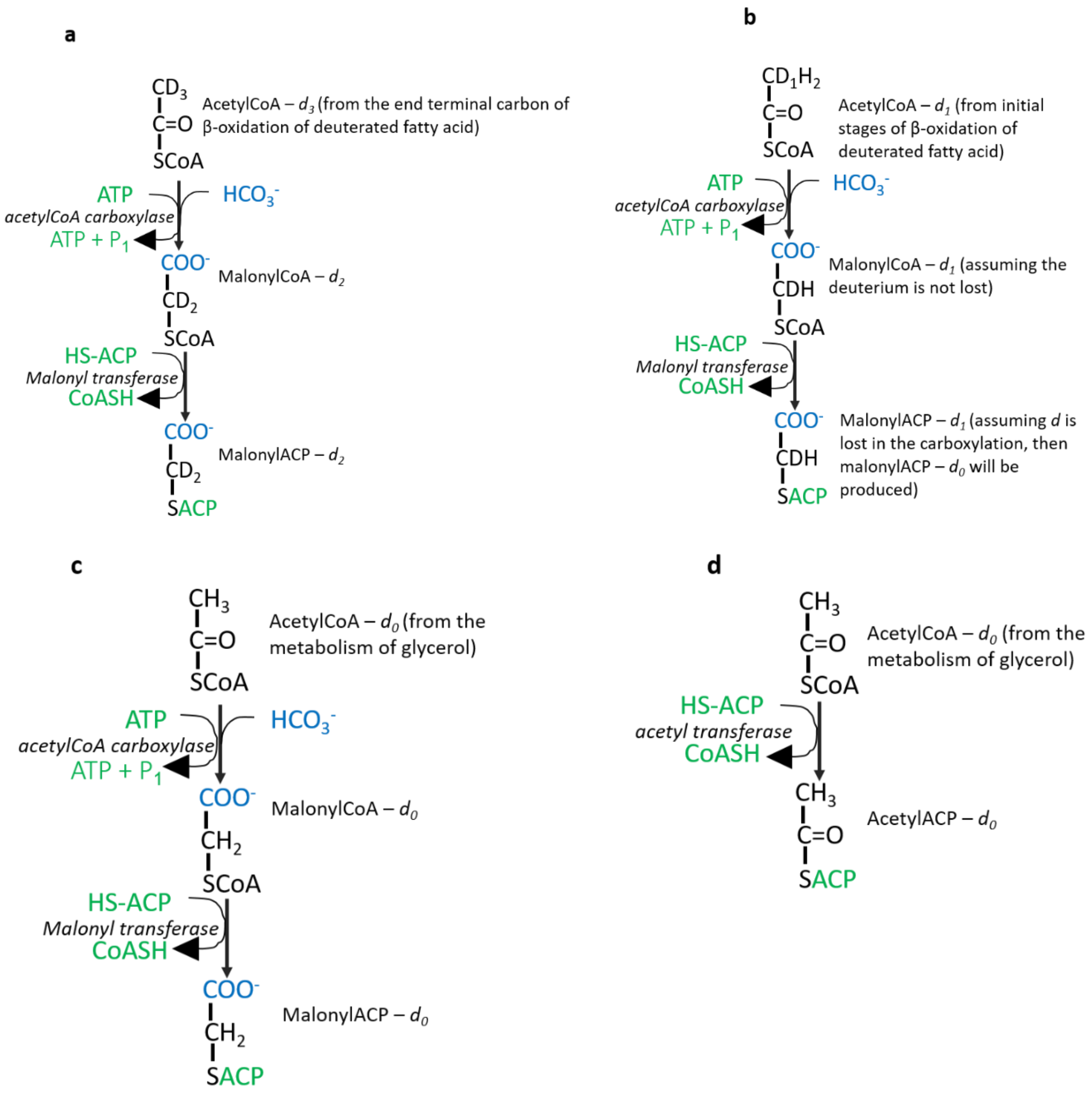

Supplement: Supplementary file 1 — (PDF 484kb) [file 253_2018_9059_MOESM1_ESM.pdf]
